# Supplementary material for: Characterizing the pharmacological interaction of the antimalarial combination artefenomel-piperaquine in healthy volunteers with induced blood-stage Plasmodium falciparum to predict efficacy in patients with malaria
Source: BMC Med. 2024 Nov 28;22:563. doi: 10.1186/s12916-024-03787-0 (PMC11603672; doi:10.1186/s12916-024-03787-0)
Supplement: Supplementary file 2 — Additional file 2: Table S1. Schedule of study activities and procedures. Table S2. Volunteer infection study data used for pharmacokinetic/ pharmacodynamic modelling of artefenomel-piperaquine combination. Table S3. Phase 2b study data used in simulations to predict APR28 in patients. Table S4. Plasma artefenomel and piperaquine non-compartmental pharmacokinetic parameters. Table S5. Individual participant parasite clearance parameters. Table S6. Parameter estimates for the final pharmacokinetic combination model of artefenomel and piperaquine from monotherapy and combination therapy volunteer infection study data. Table S7. Parameter estimates of the pharmacokinetic/pharmacodynamic model for artefenomel and piperaquine in monotherapy and in combination. Table S8. Adverse events by system organ class and preferred term. [file 12916_2024_3787_MOESM2_ESM.docx]

**Table S1. Schedule of study activities and procedures**

| **Procedure** | **Screening**  D-28 to D-1 | **Eligibility visit^a^**  D-3 to D-1 | **Malaria inoculation day**  D0 | **Post-inoculation phone contact**  D1 to D3 | **Malaria monitoring^b^**  D4 to D7 | **ART and PQP treatment and clinical unit confinement**  D8 to D11 | **Out-patient monitoring** D12 to D42±2 | **Rescue treatment with Riamet®** D42±2  or earlier if required | **EOS visit**  D45±2 |
| --- | --- | --- | --- | --- | --- | --- | --- | --- | --- |
| Informed consent & BDI | X |  |  |  |  |  |  |  |  |
| Medical history, eligibility & prior medications | X |  | X |  |  |  |  |  |  |
| Drug & alcohol screen | X |  | X |  |  | X |  |  |  |
| Full physical examination | X |  |  |  |  |  |  |  | X |
| Abbreviated physical examination |  |  | X |  |  | X^r^ |  |  |  |
| Symptom-directed physical examination^c^ |  |  |  |  | X | X | X | X |  |
| Vital sign assessment | X |  | X |  | X | X | X | X | X |
| ECG | X |  | X |  |  | X^j^ |  | X | X |
| Urinalysis | X | X |  |  |  | X^k^ | X |  | X |
| Haematology & biochemistry | X | X |  |  |  | X | X | X | X |
| Coagulation profile ^e^ | X |  |  |  |  |  |  |  |  |
| G6PD testing | X |  |  |  |  |  |  |  |  |
| RBC alloantibody | X |  |  |  |  |  |  |  | X |
| Serology | X |  |  |  |  |  |  |  | X |
| Serum β-hCG pregnancy test | X^d^ | X |  |  |  |  |  |  | X |
| Urine β-hCG pregnancy test |  |  | X |  |  | X |  |  |  |
| Safety serum storage |  |  | X |  |  |  |  |  | X |
| AEs & concomitant medications |  |  | X | X | X | X | X | X | X |
| Malaria clinical score |  |  | X |  | X | X | X^l^ | X | X |
| Malaria 18S qPCR blood sampling |  |  | X |  | X | X | X^m^ | X | X |
| Parasite lifecycle stage qRT-PCR blood sampling^f^ |  |  |  |  |  |  | X | X | X |
| ART and PQP concentration blood sampling |  |  |  |  |  | X | X | X^s^ |  |
| Phone call or text message |  |  |  |  |  |  |  | X^n^ |  |
| Malaria inoculum |  |  | X^g^ |  |  |  |  |  |  |
| ART and PQP treatment |  |  |  |  |  | X |  |  |  |
| Riamet treatment^h^ |  |  |  |  |  |  |  | X |  |
| Primacin treatment^i^ |  |  |  |  |  |  |  | X |  |
| Immune cell characterisation (optional) |  |  | X^o^ |  | X^P^ | X^q^ | X |  | X |
| Complement regulatory proteins (optional) |  |  | X^o^ |  |  | X^q^ | X |  | X |

ART: artefenomel; PQP: piperaquine phosphate; EOS: End of Study, BDI: Beck Depression Inventory; ECG: electrocardiogram; G6PD: glucose-6-phosphate dehydrogenase, RBC: red blood cell, PK: pharmacokinetic, PD: pharmacodynamic, qPCR: quantitative polymerase chain reaction, qRT-PCR: reverse transcription qPCR.

^a^ An additional safety visit occurred between Day -3 and Day -1 to collect samples for haematology, biochemistry and urinalysis, unless screening laboratory assessments were conducted within this period.

^b^ Daily visits until qPCR positive, and then twice daily visits until OZ439 and PQP treatment.

^c^ Symptom-directed physical examinations were performed only if clinically indicated at the discretion of the Investigator.

^d^ Perform follicle stimulating hormone test (post-menopausal females) at screening.

^e^ Performed at screening visit.

^f^ Performed at the Investigator’s discretion.

^g^ Administer the malaria inoculum of ~2 800 viable *P. falciparum* 3D7 infected human RBCs intravenously.

^h^ Riamet treatment occurred on Day 42±2, or earlier if there was failure of clearance defined as failure to clear parasitaemia by at least 10-fold at 72 hours post-IMP administration or recrudescence of parasitaemia (defined as ≥5 000 blood stage parasites/mL and a 2-fold increase within 48 hours, or a malaria clinical score >6), or at the Investigator’s discretion.

^i^ Primacin treatment if required.

^j^ ECG performed in triplicate at 4, 6, 8, 12, 24 and 72 hours post-ART and PQP dosing, and prior to Riamet treatment or at the Investigator’s discretion.

^k^ At the time of admission to clinical unit only.

^l^ Only if vital signs were abnormal, or at the Investigator’s discretion.

^m^ Malaria 18S qPCR performed Day 12 (am & pm), Day 13 (am & pm) then 3x per week until EOS at Investigators discretion.

^n^ Phone call for 3 days to ensure adherence to Riamet treatment.

^o^ Blood collection pre-inoculation.

^p^ Blood collection Day 4 visit.

^q^ Blood collection pre-ART and PQP dosing.

^r^ Abbreviated physical examination pre-ART and PQP dosing.

^s^ Blood collection for ART and PQP PK at Day 42±2. If Riamet treatment occurred prior to Day 42±2 the PK collection continued as per scheduled time-points.

**Table S2.** **Volunteer infection study (VIS) data used for pharmacokinetic/ pharmacodynamic modelling of artefenomel-piperaquine combination**

| **Study** | **Dose** | **Number of participants** |
| --- | --- | --- |
| McCarthy et al., 2016 [reference 8] | Artefenomel 100 mg  Artefenomel 200 mg  Artefenomel 500 mg | 8  8  8 |
| Pasay et al., 2016 [reference 14] | Piperaquine 480 mg  Piperaquine 640 mg  Piperaquine 960 mg | 12^a^  7  5 |
| Abd-Rahman et al. [current study] | Artefenomel 200 mg/ piperaquine 320 mg  Artefenomel 200 mg/ piperaquine 480 mg  Artefenomel 200 mg/ piperaquine 640 mg  Artefenomel 400 mg/ piperaquine 480 mg  Artefenomel 400 mg/ piperaquine 640 mg  Artefenomel 800 mg/ piperaquine 640 mg  Artefenomel 800 mg/ piperaquine 960 mg | 4  2  2  2  6  4  4 |
| TOTAL | | 72 |

^a^Nine participants initially dosed with 480 mg piperaquine received a second dose of piperaquine (960 mg).

**Table S3. Phase 2b study data used in simulations to predict APR_28_ in patients**

| **Study** | **Dose** | **Number of participants^a^** | **Age (years)^c^** | **Weight (kg)^c^** | **Baseline parasitaemia (parasites/μL)^c^** |
| --- | --- | --- | --- | --- | --- |
| Macintyre et al., 2017 [reference 13] | ART 800 mg/ PQP 640 mg^b^  ART 800 mg/ PQP 960 mg^b^  ART 800 mg/ PQP 1440 mg^b^ | 108  119  119 | 11.19 (0.5-57) | 27.45 (5.6-77.2) | 25718 (187-220240) |

^a^Number of participants with pharmacokinetic data. ^b^Nominal doses for children scaled to achieve similar exposure. ^c^Mean values for all dose groups with range.

**Table S4. Plasma artefenomel and piperaquine non-compartmental pharmacokinetic parameters**

| **Parameter** | **Artefenomel 200 mg/ piperaquine 480 mg**  **[N=2]** | **Artefenomel 200 mg/ piperaquine 640 mg**  **[N=2]** | **Artefenomel 400 mg/ piperaquine 480 mg**  **[N=2]** | **Artefenomel 400 mg/ piperaquine 640 mg**  **[N=6]** | **Artefenomel 800 mg/ piperaquine 960 mg**  **[N=4]** | **Artefenomel 200 mg/ piperaquine 320 mg**  **[N=4]** | **Artefenomel 800 mg/ piperaquine 640 mg**  **[N=4]** |
| --- | --- | --- | --- | --- | --- | --- | --- |
| **Artefenomel** | | | | | | | |
| C_max_ (ng/L) | 0.4145 (41.4) | 0.2582 (22.9) | 0.8829 (48.2) | 0.6835 (32.7) | 2.135 (38.0) | 0.3208 (113.6) | 1.910 (15.3) |
| t_max_ (h) | 2.5 (2.0,3.0) | 2.5 (2.0,3.0) | 3.0 (3.0,3.0) | 2.5 (2.0,3.0) | 2.5 (2.0,3.0) | 2.0 (2.0,6.0) | 3.0 (3.0,3.0) |
| AUC_0-last_ (h*ng/L) | 2.861 (91.4) | 1.763 (27.0) | 8.734 (60.7) | 6.245 (25.5) | 20.29 (32.4) | 1.582 (72.7) | 19.39 (6.8) |
| AUC_0-inf_ (h*ng/L) | NR | NR | 9.056 (57.3) | 6.702 (29.9) | 23.84 (13.1)^b^ | NR | 19.78 (6.6) |
| t_1/2_ (h) | NR | NR | 146.4 (2.9) | 147.1 (68.8) | 161.5 (25.8)^b^ | NR | 168.4 (49.0) |
| CL/F (L/h) | NR | NR | 44.17 (57.3) | 59.68 (29.9) | 33.55 (13.1)^b^ | NR | 40.44 (6.6) |
| Vz/F (L) | NR | NR | 9330 (53.7) | 12660 (39.8) | 7818 (33.0)^b^ | NR | 9825 (52.3) |
| **Piperaquine** | | | | | | | |
| C_max_ (ng/L) | 0.05441 (39.0) | 0.1168 (31.3) | 0.06458 (45.9) | 0.1048 (99.6) | 0.4104 (19.7) | 0.04609 (124.2) | 0.1860 (100.1) |
| t_max_ (h) | 4.0 (3.0,5.0) | 5.0 (4.0,6.0) | 3.5 (3.0,4.0) | 4.0 (2.0, 16.0) | 3.0 (2.0,4.0) | 4.0 (2.0,12.2) | 4.5 (2.0,24.0) |
| AUC_0-last_ (h*ng/L) | 3.778 (0.5) | 8.760 (15.8) | 3.575 (50.5) | 5.794 (30.0) | 16.14 (6.1) | 2.386 (60.6) | 10.49 (43.8) |
| AUC_0-inf_ (h*ng/L) | 4.257 (3.3) | NR | NR | 8.169 (ND)^a^ | 20.25 (0.7)^c^ | 3.803 (17.0)^c^ | 16.70 (17.1)^c^ |
| t_1/2_ (h) | 262.3 (12.7) | NR | NR | 393.6 (ND)^a^ | 395.9 (6.8)^c^ | 267.2 (25.8)^c^ | 253.2 (8.9)^c^ |
| CL/F (L/h) | 112.8 (3.3) | NR | NR | 78.35 ND)^a^ | 47.42 (0.7)^c^ | 84.15 (17.0)^c^ | 38.32 (17.1)^c^ |
| Vz/F (L) | 42670 (9.4) | NR | NR | 44480 (ND)^a^ | 27080 (6.1)^c^ | 32430 (8.6)^c^ | 14000 (26.3)^c^ |

Data are geometric means (coefficient of variation [%]) except t_max_ which is median (minimum, maximum). C_max_: maximum observed concentration; t_max_: time to reach the maximum observed concentration; AUC_0-last_: area under the concentration-time curve from time 0 (dosing) to the last sampling time at which the concentration is at or above the lower limit of quantification; AUC_0-inf_: area under the concentration-time curve from time 0 (dosing) extrapolated to infinity; t_½_: apparent terminal half-life; CL/F: apparent total clearance; Vz/F: apparent volume of distribution; NR: not reported; ND: not determined. Calculation based on n=1^a^, n=3^b^, or n=2^c^. Values for AUC_0-inf_, t_1/2_, CL/F, and Vz/F were only reported for a participant if the following criteria were met: a minimum of 3 measurable concentration-time points during the log-linear portion of the terminal elimination phase (excluding C_max_); r^2^ > 0.80 for the regression of the log concentration-time data during the terminal elimination phase; negative slope for log regression fit; extrapolated portion of AUC_0-inf_ < 20% of total AUC_0-inf_.

**Table S5. Individual participant parasite clearance parameters**

| **Participant ID** | **Log_10_PRR_48_ (95% CI)** | **PCt_1/2_ (95% CI)** | **Parasite regrowth*** |
| --- | --- | --- | --- |
| **Artefenomel 200 mg/ piperaquine 480 mg** | | |  |
| 103 | 2.3 (2.07 - 2.52) | 6.29 (5.73 - 6.97) | Yes (day 22) |
| 106 | 1.98 (1.69 - 2.27) | 7.29 (6.36 - 8.55) | Yes (day 12) |
| **Artefenomel 200 mg/ piperaquine 640 mg** | | | |
| 102 | 2.17 (1.88 - 2.45) | 6.67 (5.89 - 7.69) | No |
| 104 | 3.01 (2.44 - 3.57) | 4.8 (4.04 - 5.91) | No |
| **Artefenomel 400 mg/ piperaquine 480 mg** | | | |
| 101 | 3.28 (2.96 - 3.59) | 4.41 (4.02 - 4.88) | No |
| 108 | 4.84 (4.2 - 5.49) | 2.98 (2.63 - 3.44) | Yes (day 22) |
| **Artefenomel 400 mg/ piperaquine 640 mg** | | |  |
| 105 | 3.39 (2.85 - 3.93) | 4.26 (3.67 - 5.07) | No |
| 107 | 3.3 (2.9 - 3.7) | 4.38 (3.9 - 4.99) | No |
| 302 | 3.33 (2.96 - 3.69) | 4.34 (3.91 - 4.88) | No |
| 306 | 4.17 (3.68 - 4.66) | 3.46 (3.1 - 3.93) | No |
| 307 | 5.66 (5.03 - 6.28) | 2.55 (2.3 - 2.87) | No |
| 308 | 3.62 (3.26 - 3.98) | 3.99 (3.63 - 4.43) | No |
| **Artefenomel 800 mg/ piperaquine 960 mg** | | | |
| 201 | 2.81 (2.31 - 3.31) | 5.14 (4.37 - 6.24) | No |
| 205 | 4.32 (3.98 - 4.67) | 3.34 (3.1 - 3.63) | No |
| 206 | 4.52 (3.37 - 5.67) | 3.2 (2.55 - 4.29) | No |
| 207 | 4.5 (3.91 - 5.09) | 3.21 (2.84 - 3.7) | No |
| **Artefenomel 200 mg/ piperaquine 320 mg** | | |  |
| 202 | 3.13 (2.84 - 3.42) | 4.62 (4.22 - 5.09) | Yes (day 13) |
| 203 | 1.2 (0.96 - 1.44) | 12.04 (10.06 - 14.99) | Yes (day 13) |
| 204 | 2.19 (1.83 - 2.56) | 6.59 (5.65 - 7.89) | Yes (day 13) |
| 208 | 1.56 (1.28 - 1.84) | 9.24 (7.84 - 11.26) | Yes (day 13) |
| **Artefenomel 800 mg/ piperaquine 640 mg** | | | |
| 301 | 3.46 (3.01 - 3.92) | 4.17 (3.69 - 4.81) | No |
| 303 | 3.38 (2.99 - 3.77) | 4.28 (3.84 - 4.84) | No |
| 304 | 4.88 (4.15 - 5.61) | 2.96 (2.58 - 3.48) | No |
| 305 | 3.49 (3.2 - 3.79) | 4.14 (3.82 - 4.52) | No |

Log_10_PRR_48_: parasite reduction ration over a 48 hour period on a log scale; PCt_1/2_: parasite clearance half-life in hours; CI: confidence interval. *The day of parasite regrowth is defined as the day prior to the day on which artemether-lumefantrine treatment was initiated (day relative to inoculation with the malaria challenge agent).

**Table S6. Parameter estimates for the final pharmacokinetic combination model of artefenomel and piperaquine from monotherapy and combination therapy volunteer infection study data**

| PARAMETER | VALUE | RSE | SHRINKAGE | COMMENT |
| --- | --- | --- | --- | --- |
| **Typical parameters** |  |  |  |  |
| Fabs1x1 | 1 (FIX) | - | - | Relative bioavailability artefenomel (-) |
| kax1 | 0.216 | 4.05% | - | Absorption rate parameter artefenomel (1/hour) |
| CLx1 | 68 | 5.97% | - | Apparent clearance artefenomel (L/hour) |
| Vcx1 | 81.5 | 12.2% | - | Apparent central volume artefenomel (L) |
| Q1x1 | 10.6 | 4.32% | - | Apparent intercompartmental clearance artefenomel (L/hour) |
| Vp1x1 | 1070 | 7.61% | - | Apparent peripheral volume artefenomel (L) |
| Fabs1x2 | 1 (FIX) | - | - | Relative bioavailability piperaquine (-) |
| kax2 | 0.0794 | 9.32% | - | Absorption rate parameter piperaquine (1/hour) |
| CLx2 | 72.3 | 7.28% | - | Apparent clearance piperaquine (L/hour) |
| Vcx2 | 582 | 11.8% | - | Apparent central volume piperaquine (L) |
| Q1x2 | 334 | 7.38% | - | Apparent intercompartmental clearance piperaquine (L/hour) |
| Vp1x2 | 30800 | 8.11% | - | Apparent peripheral volume piperaquine (L) |
| Tlag1 | 0.409 | 2.18% | - | - |
| Tlag2 | 0.39 | 2.8% | - | - |
|  |  |  |  |  |
| **Inter-individual variability** |  |  |  |  |
| omega(Fabs1x1) | 0 (FIX) | - | - | Normal |
| omega(kax1) | 0.0897 | 42.2% | 61% | LogNormal |
| omega(CLx1) | 0.348 | 11.3% | 21% | LogNormal |
| omega(Vcx1) | 0.687 | 12.8% | 29% | LogNormal |
| omega(Q1x1) | 0.05 (FIX) | - | 74% | LogNormal |
| omega(Vp1x1) | 0.572 | 21.9% | 24% | LogNormal |
| omega(Fabs1x2) | 0 (FIX) | - | - | Normal |
| omega(kax2) | 0.495 | 13.4% | 27% | LogNormal |
| omega(CLx2) | 0.461 | 11.9% | 22% | LogNormal |
| omega(Vcx2) | 0.463 | 23.2% | 38% | LogNormal |
| omega(Q1x2) | 0.409 | 14.1% | 31% | LogNormal |
| omega(Vp1x2) | 0.505 | 12.5% | 25% | LogNormal |
| omega(Tlag1) | 0.104 | 17.6% | 31% | LogNormal |
| omega(Tlag2) | 0.144 | 13.6% | 29% | LogNormal |
|  |  |  |  |  |
| **Correlation of random effects** |  |  |  |  |
| corr(CLx1,Vcx1) | -0.148 | 110% | - | Correlation coefficient |
| corr(CLx2,Vcx2) | 0.577 | 33.9% | - | Correlation coefficient |
|  |  |  |  |  |
| **Parameter-Covariate relationships** |  |  |  |  |
| beta_kax1(AUC1) | -0.377 | 11.6% | - | AUC of artefenomel in mg/kg^0.75 on kax1 |
| beta_CLx1(AUC1) | -0.383 | 16.7% | - | AUC of artefenomel in mg/kg^0.75 on CLx1 |
| beta_CLx1(WT0) | 0.75 (FIX) | - | - | Weight in kg on CLx1 |
| beta_Vcx1(WT0) | 1 (FIX) | - | - | Weight in kg on Vcx1 |
| beta_Q1x1(WT0) | 0.75 (FIX) | - | - | Weight in kg on Q1x1 |
| beta_Vp1x1(WT0) | 1 (FIX) | - | - | Weight in kg on Vp1x1 |
| beta_CLx2(WT0) | 0.75 (FIX) | - | - | Weight in kg on CLx2 |
| beta_Vcx2(WT0) | 1 (FIX) | - | - | Weight in kg on Vcx2 |
| beta_Q1x2(WT0) | 0.75 (FIX) | - | - | Weight in kg on Q1x2 |
| beta_Vp1x2(WT0) | 1 (FIX) | - | - | Weight in kg on Vp1x2 |
|  |  |  |  |  |
| **Residual Variability** |  |  |  |  |
| error_PROP1 | 0.354 | 2.67% | - | Proportional Error (fraction) - Concentration artefenomel |
| error_PROP2 | 0.366 | 2.68% | - | Proportional Error (fraction) - Concentration piperaquine |
|  |  |  |  |  |
| Objective function | -9757 | - | - | - |
| AIC | -9699 | - | - | - |
| BIC | -9633 | - | - | - |

Objective function rounded to closest integer value, omega values reported in standard deviation.

**Table S7. Parameter estimates of the pharmacokinetic/pharmacodynamic model for artefenomel and piperaquine in monotherapy and in combination**

| **Parameters** | **Description** | **Estimates^a^** |
| --- | --- | --- |
| *Natural growth* | | |
| PL_base_ (ln parasites) | Baseline parasitemia | -3.64 (19) |
| k_grow_ (/h) | Growth rate constant | 0.0709 (6.22) |
| IIV PL_base_ | Interindividual variability in PL_base_ | 2.5 (17)^b^ |
| IIV k_grow_ | Interindividual variability in k_grow_ | 0.221 (15.4)^b^ |
| *Artefenomel exposure-effect relationship monotherapy* | | |
| EMAXx1 (/h) | Maximum killing rate of artefenomel | 0.194 (10.3) |
| EC50x1 (ng/mL) | Artefenomel concentration at which half EMAXx1 is reached | 2.89 (26.7) |
| hillx1 | Steepness of the concentration-effect relationship | 3.09 (6.35) |
| *Piperaquine exposure-effect relationship monotherapy* | | |
| EMAXx2 (/h) | Maximum killing rate of piperaquine | 0.262 (3.82) |
| EC50x2 (ng/mL) | Piperaquine concentration at which half EMAXx2 is reached | 6.26 (17.9) |
| hillx2 | Steepness of the concentration-effect relationship | 5.24 (5.32) |
| *Pharmacodynamic interaction (GPDI model)* | | |
| INT_EC50_ | Maximal fractional change of EC_50_ | -0.117 (7.12) |
| INT_Emax_ | Maximal fractional change of E_max_ | -0.406 (8.19) |
| EC50_INT12, EC50_ (ng/mL) | Potency of the EC_50_ interaction of artefenomel mediated by piperaquine | 6.26 (fixed) |
| EC50_INT21, EC50_ (ng/mL) | Potency of the EC_50_ interaction of piperaquine mediated by artefenomel | 0.196 (fixed) |
| EC50_INT12, Emax_ (ng/mL) | Potency of the E_max_ interaction of artefenomel mediated by piperaquine | 6.26 (fixed) |
| EC50_INT21, Emax_ (ng/mL) | Potency of the E_max_ interaction of piperaquine mediated by artefenomel | 0.196 (fixed) |
| *Residual error* | | |
| Proportional error |  | 1.29 (3.4) |

^a^ Value in estimate (relative standard error) unless otherwise stated.

^b^ Value in standard deviation (relative standard error).

**Table S8. Adverse events by system organ class and preferred term**

| **System organ class**  Preferred term | **Overall (N=24)** | **Artefenomel 200 mg/  piperaquine 480 mg (N=2)** | **Artefenomel 200 mg/ piperaquine 640 mg (N=2)** | **Artefenomel 400 mg/ piperaquine 480 mg (N=2)** | **Artefenomel 400 mg/ piperaquine 640 mg (N=6)** | **Artefenomel 800 mg/ piperaquine 960 mg (N=4)** | **Artefenomel 200 mg/ piperaquine 320 mg (N=4)** | **Artefenomel 800 mg/ piperaquine 640 mg (N=4)** |
| --- | --- | --- | --- | --- | --- | --- | --- | --- |
|  | **Number of participants with adverse event (%) Number of adverse events** | | | | | | | |
| Any adverse event | 21 (87.5) 101 | 2 (100) 10 | 2 (100) 3 | 2 (100) 14 | 4 (66.7) 16 | 4 (100) 22 | 3 (75.0) 19 | 4 (100) 17 |
| **Nervous system disorders** | **17 (70.8) 19** | **2 (100%) 2** | **0** | **2 (100) 2** | **3 (50.0) 4** | **4 (100) 5** | **3 (75.0) 3** | **3 (75.0) 3** |
| Headache | 15 (62.5) 15 | 1 (50.0) 1 | 0 | 2 (100) 2 | 3 (50.0) 3 | 4 (100) 4 | 3 (75.0) 3 | 2 (50.0) 2 |
| Dizziness | 2 (8.3) 2 | 1 (50.0) 1 | 0 | 0 | 0 | 0 | 0 | 1 (25.0) 1 |
| Hypoaesthesia | 1 (4.2) 1 | 0 | 0 | 0 | 0 | 1 (25.0) 1 | 0 | 0 |
| Lethargy | 1 (4.2) 1 | 0 | 0 | 0 | 1 (16.7) 1 | 0 | 0 | 0 |
| **General disorders and administration site conditions** | **16 (66.7) 25** | **2 (100) 5** | **2 (100) 2** | **1 (50.0) 4** | **3 (50.0) 5** | **2 (50.0) 2** | **3 (75.0) 4** | **3 (75.0) 3** |
| Fatigue | 8 (33.3) 8 | 1 (50.0) 1 | 1 (50.0) 1 | 1 (50.0) 1 | 1 (16.7) 1 | 0 | 1 (25.0) 1 | 3 (75.0) 3 |
| Pyrexia | 7 (29.2) 7 | 2 (100) 2 | 0 | 1 (50.0) 1 | 2 (33.3) 2 | 0 | 2 (50.0) 2 | 0 |
| Vessel puncture site bruise | 5 (20.8) 5 | 0 | 1 (50.0) 1 | 1 (50.0) 1 | 1 (16.7) 1 | 2 (50.0) 2 | 0 | 0 |
| Chills | 3 (12.5) 3 | 1 (50.0) 1 | 0 | 0 | 1 (16.7) 1 | 0 | 1 (25.0) 1 | 0 |
| Feeling hot | 1 (4.2) 1 | 1 (50.0) 1 | 0 | 0 | 0 | 0 | 0 | 0 |
| Malaise | 1 (4.2) 1 | 0 | 0 | 1 (50.0) 1 | 0 | 0 | 0 | 0 |
| **Musculoskeletal and connective tissue disorders** | **12 (50.0) 17** | **1 (50.0) 1** | **1 (50.0) 1** | **2 (100) 3** | **2 (33.3) 3** | **2 (50.0) 3** | **2 (50.0) 4** | **2 (50.0) 2** |
| Myalgia | 8 (33.3) 8 | 1 (50.0) 1 | 0 | 2 (100) 2 | 1 (16.7) 1 | 1 (25.0) 1 | 2 (50.0) 2 | 1 (25.0) 1 |
| Back pain | 5 (20.8) 5 | 0 | 0 | 0 | 1 (16.7) 1 | 2 (50.0) 2 | 1 (25.0) 1 | 1 (25.0) 1 |
| Arthralgia | 2 (8.3) 2 | 0 | 0 | 1 (50.0) 1 | 1 (16.7) 1 | 0 | 0 | 0 |
| Neck pain | 1 (4.2) 1 | 0 | 0 | 0 | 0 | 0 | 1 (25.0) 1 | 0 |
| Pain in extremity | 1 (4.2) 1 | 0 | 1 (50.0) 1 | 0 | 0 | 0 | 0 | 0 |
| **Gastrointestinal disorders** | **10 (41.7) 11** | **1 (50.0) 1** | **0** | **2 (100) 3** | **0** | **4 (100) 4** | **1 (25.0) 1** | **2 (50.0) 2** |
| Nausea | 10 (41.7) 10 | 1 (50.0) 1 | 0 | 2 (100) 2 | 0 | 4 (100) 4 | 1 (25.0) 1 | 2 (50.0) 2 |
| Abdominal discomfort | 1 (4.2) 1 | 0 | 0 | 1 (50.0) 1 | 0 | 0 | 0 | 0 |
| **Investigations** | **9 (37.5) 12** | **0** | **0** | **2 (100) 2** | **1 (16.7) 1** | **1 (25.0) 1** | **3 (75.0) 5** | **2 (50.0) 3** |
| Fall in haemoglobin | 4 (16.7) 4 | 0 | 0 | 1 (50.0) 1 | 0 | 0 | 1 (25.0) 1 | 2 (50.0) 2 |
| Fall in white blood cell count | 1 (4.2) 1 | 0 | 0 | 0 | 0 | 0 | 1 (25.0) 1 | 0 |
| Fall in lymphocyte count | 4 (16.7) 4 | 0 | 0 | 1 (50.0) 1 | 0 | 0 | 2 (50.0) 2 | 1 (25.0) 1 |
| Fall in neutrophil count | 2 (8.3) 2 | 0 | 0 | 0 | 1 (16.7) 1 | 0 | 1 (25.0) 1 | 0 |
| Prolongation of QT interval | 1 (4.2) 1 | 0 | 0 | 0 | 0 | 1 (25.0) 1 | 0 | 0 |
| **Injury, poisoning and procedural complications** | **5 (20.8) 5** | **0** | **0** | **0** | **1 (16.7) 1** | **3 (75.0) 3** | **1 (25.0) 1** | **0** |
| Contusion | 2 (8.3%) 2 | 0 | 0 | 0 | 0 | 2 (50.0) 2 | 0 | 0 |
| Sunburn | 1 (4.2) 1 | 0 | 0 | 0 | 0 | 1 (25.0) 1 | 0 | 0 |
| Vascular access site bruising | 1 (4.2) 1 | 0 | 0 | 0 | 1 (16.7) 1 | 0 | 0 | 0 |
| Wrist fracture | 1 (4.2) 1 | 0 | 0 | 0 | 0 | 0 | 1 (25.0) 1 | 0 |
| **Infections and infestations** | **4 (16.7) 4** | **0** | **0** | **0** | **0** | **1 (25.0) 1** | **1 (25.0) 1** | **2 (50.0) 2** |
| Upper respiratory tract infection | 2 (8.3) 2 | 0 | 0 | 0 | 0 | 0 | 0 | 2 (50.0) 2 |
| Rhinovirus infection | 1 (4.2) 1 | 0 | 0 | 0 | 0 | 0 | 1 (25.0) 1 | 0 |
| Viral infection | 1 (4.2) 1 | 0 | 0 | 0 | 0 | 1 (25.0) 1 | 0 | 0 |
| **Cardiac disorders** | **2 (8.3) 2** | **1 (50.0) 1** | **0** | **0** | **0** | **0** | **0** | **1 (25.0) 1** |
| Tachycardia | 2 (8.3) 2 | 1 (50.0) 1 | 0 | 0 | 0 | 0 | 0 | 1 (25.0) 1 |
| Metabolism and nutrition disorders | 2 (8.3) 2 | 0 | 0 | 0 | 0 | 1 (25.0) 1 | 0 | 1 (25.0) 1 |
| Decreased appetite | 2 (8.3%) 2 | 0 | 0 | 0 | 0 | 1 (25.0) 1 | 0 | 1 (25.0) 1 |
| **Respiratory, thoracic and mediastinal disorders** | **2 (8.3) 2** | **0** | **0** | **0** | **1 (16.7) 1** | **1 (25.0) 1** | **0** | **0** |
| Dry throat | 1 (4.2) 1 | 0 | 0 | 0 | 0 | 1 (25.0) 1 | 0 | 0 |
| Nasal congestion | 1 (4.2) 1 | 0 | 0 | 0 | 1 (16.7) 1 | 0 | 0 | 0 |
| **Eye disorders** | **1 (4.2) 1** | **0** | **0** | **0** | **0** | **1 (25.0) 1** | **0** | **0** |
| Dry eye | 1 (4.2) 1 | 0 | 0 | 0 | 0 | 1 (25.0) 1 | 0 | 0 |
| **Skin and subcutaneous tissue disorders** | **1 (4.2) 1** | **0** | **0** | **0** | **1 (16.7) 1** | **0** | **0** | **0** |
| Contact dermatitis | 1 (4.2) 1 | 0 | 0 | 0 | 1 (16.7) 1 | 0 | 0 | 0 |

Adverse events were coded to system organ class and preferred term using MedDRA^®^ Version 21.0.
