# Supplementary material for: Characterizing the pharmacological interaction of the antimalarial combination artefenomel-piperaquine in healthy volunteers with induced blood-stage Plasmodium falciparum to predict efficacy in patients with malaria
Source: BMC Med. 2024 Nov 28;22:563. doi: 10.1186/s12916-024-03787-0 (PMC11603672; doi:10.1186/s12916-024-03787-0)
Supplement: Supplementary file 3 — Additional file 3: Figure S1. Graphical overview of the PK/PD modelling approach and simulations performed. Figure S2. Visual predictive checks for artefenomel concentration–time profiles from observed results when administered as monotherapy and simulations using the VIS PK model. Figure S3. Visual predictive checks for piperaquine concentration–time profiles from observed results when administered as monotherapy and simulations using the VIS PK model. Figure S4. Visual predictive checks for artefenomel concentration–time profiles from observed results when administered in combination with piperaquine and simulations using the VIS PK model. Figure S5. Visual predictive checks for piperaquine concentration–time profiles from observed results when administered in combination with artefenomel and simulations using the VIS PK model. Figure S6. Individual fits for participants in the artefenomel + piperaquine combination volunteer infection study. Figure S7. Artefenomel plasma concentration–time profiles by body weight of patients in the phase 2b trial compared to the PK model built from VIS data. Figure S8. Piperaquine plasma concentration–time profiles by body weight of patients in the phase 2b trial compared with the PK model built from VIS data. Figure S9. Parasite killing rate as a function of artefenomel and piperaquine concentration. [file 12916_2024_3787_MOESM3_ESM.docx]

**Figure S1. Graphical overview of the PK/PD modelling approach and simulations performed**

**PK parameters** (absorption, distribution and elimination parameters) estimated from:

- Artefenomel (ART, OZ439) monotherapy VIS
- Piperaquine (PQP) monotherapy VIS
- ART/PQP combination VIS

**PD parameters** (EMAXx1, EC50x1, Hillx1 and EMAXx2, EC50x2, Hillx2) estimated from:

- ART monotherapy VIS
- PQP monotherapy VIS

**Interaction PD parameters** (INT_EMAX_, INT_EC50_, etc) estimated from:

- ART/PQP combination VIS

**PK/PD modelling**

**PK parameters** were sampled from their distribution from VIS PK/PD model

**PK parameters** were individual PK parameters of the phase 2b patients

**PD parameters** were sampled from their distribution from VIS PK/PD model

**PD parameters** were sampled from their distribution from VIS PK/PD model

**Simulations**

**PK/PD model (VIS PK)**

**PK/PD model (Patient PK)**

Predicted individual parasitaemia profiles

**Baseline parasitemia** were actual values recorded in phase 2b patients

**Baseline parasitemia** were actual values recorded in phase 2b patients

Predicted individual parasitaemia profiles

Predicted APR_28_

Predicted APR_28_

Observed individual parasitaemia profiles

Observed APR_28_

**Phase 2b study**

Compared

Compared

**Figure S2. Visual predictive checks for artefenomel (OZ439) concentration-time profiles from observed results when administered as monotherapy and simulations using the VIS PK model**


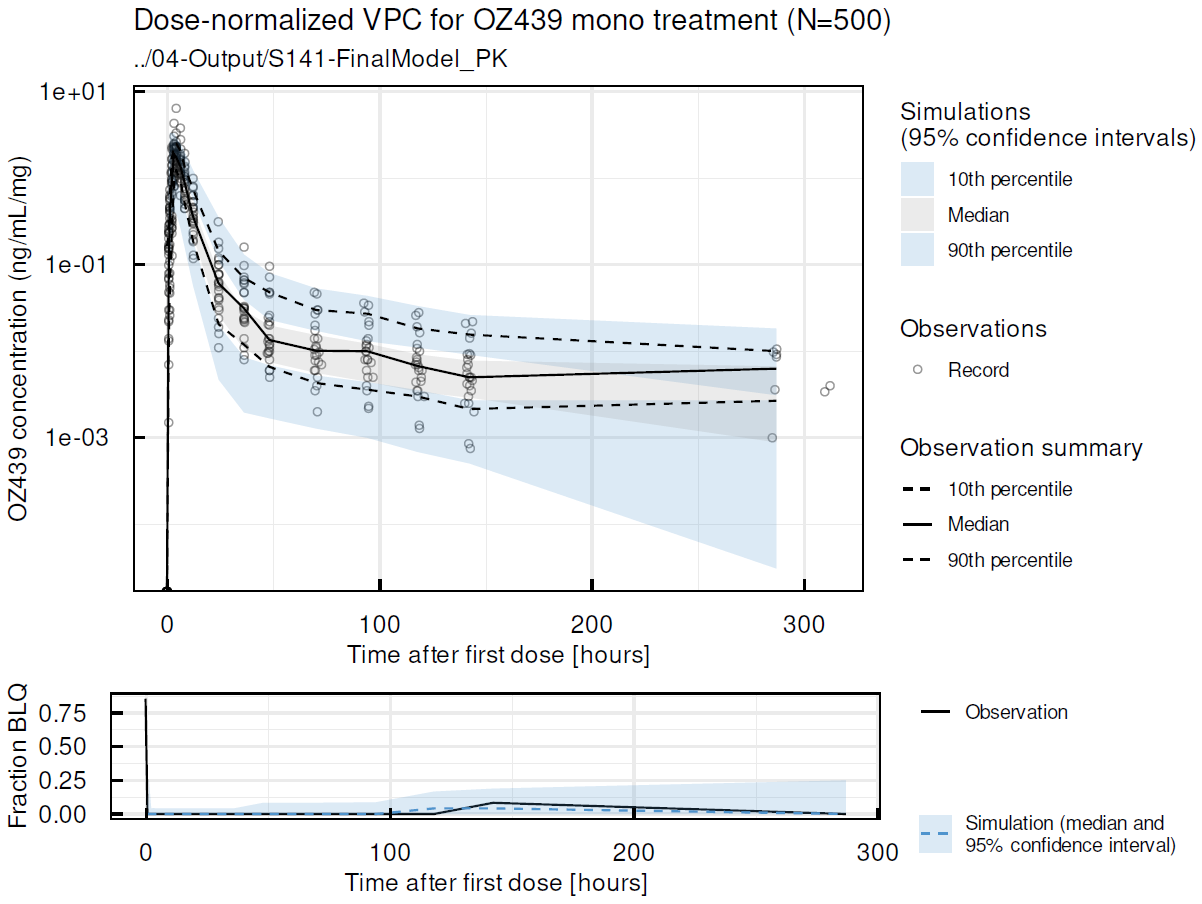


**Figure S3. Visual predictive checks for piperaquine (PQP) concentration-time profiles from observed results when administered as monotherapy and simulations using the VIS PK model**


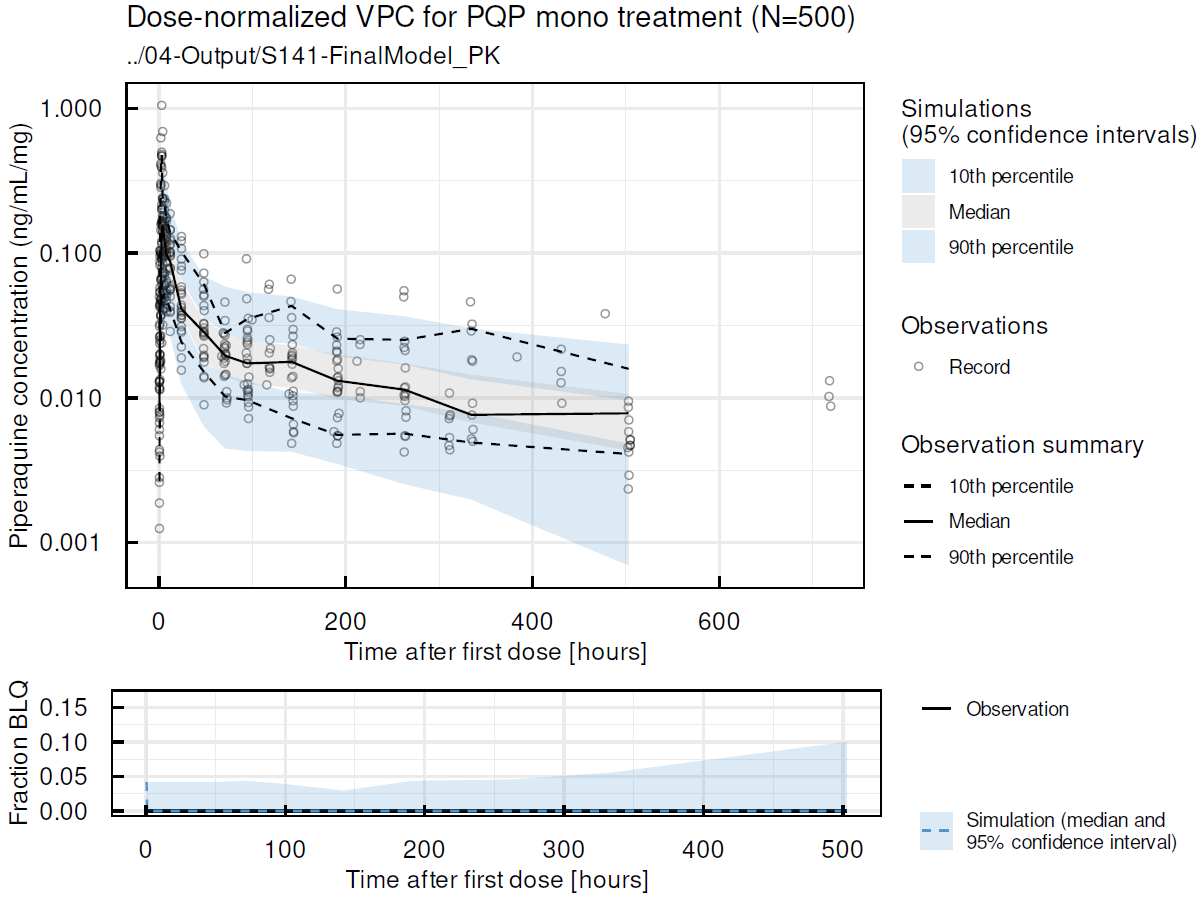


**Figure S4. Visual predictive checks for artefenomel (OZ439) concentration-time profiles from observed results when administered in combination with piperaquine (PQP) and simulations using the VIS PK model**


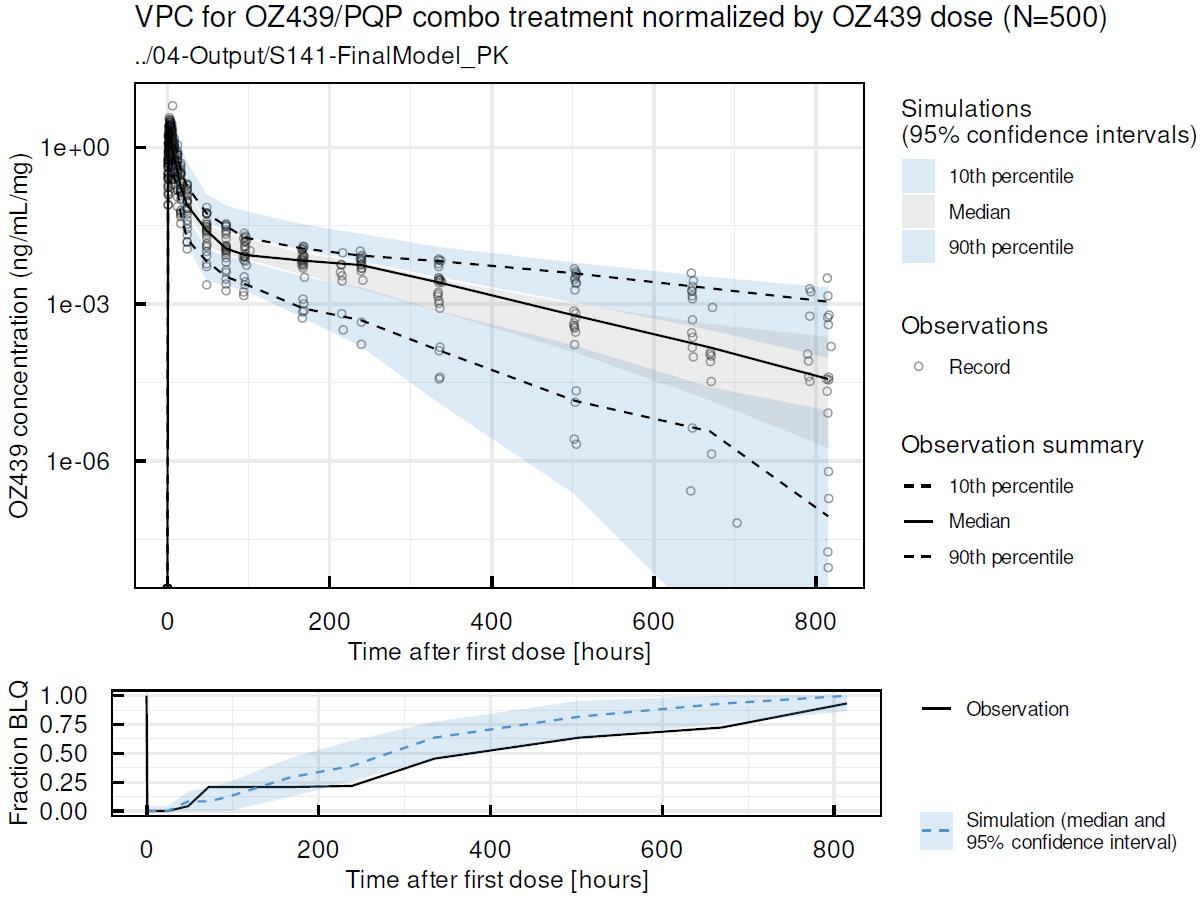


**Figure S5. Visual predictive checks for piperaquine (PQP) concentration-time profiles from observed results when administered in combination with artefenomel (OZ439) and simulations using the VIS PK model**


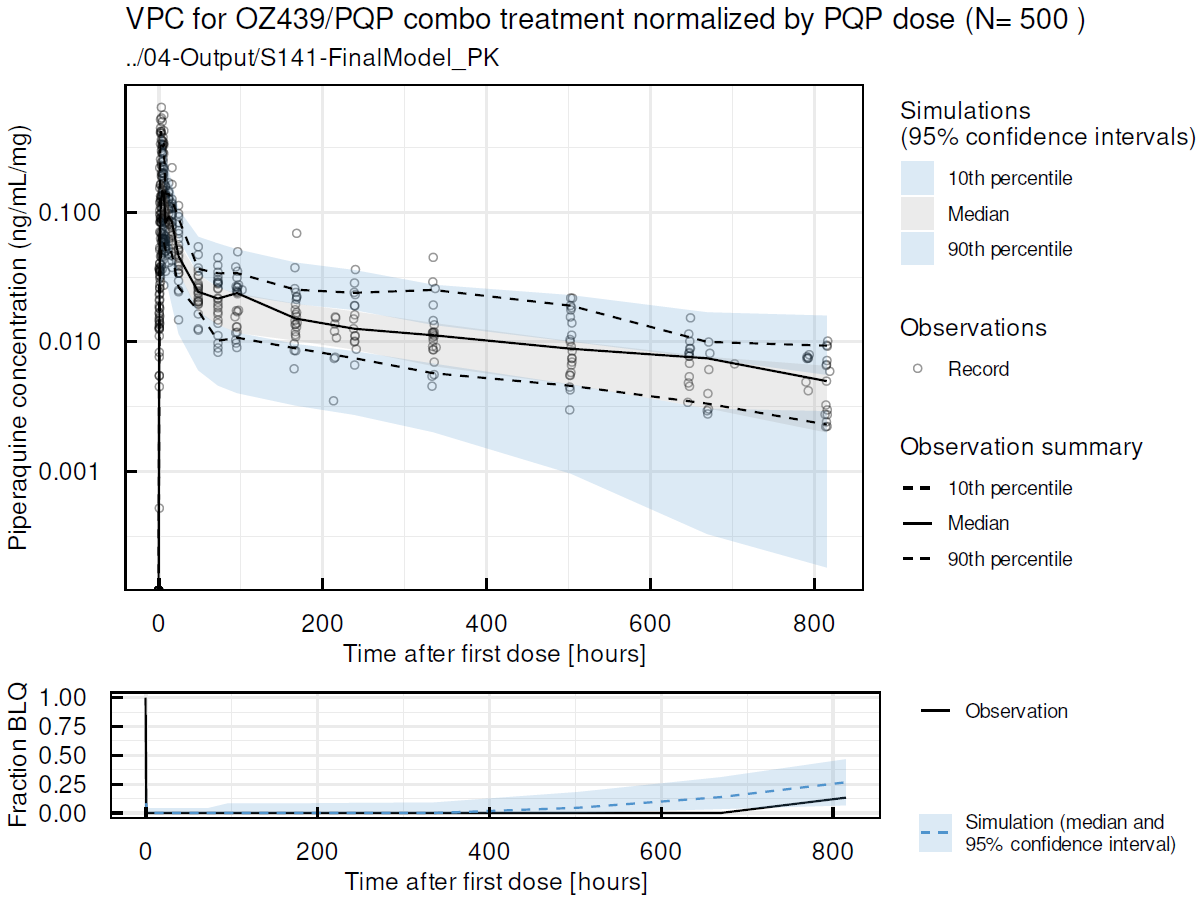


**Figure S6. Individual fits for participants in the artefenomel (OZ439) + piperaquine (PQP) combination volunteer infection study (GPDI model)**

**A.**


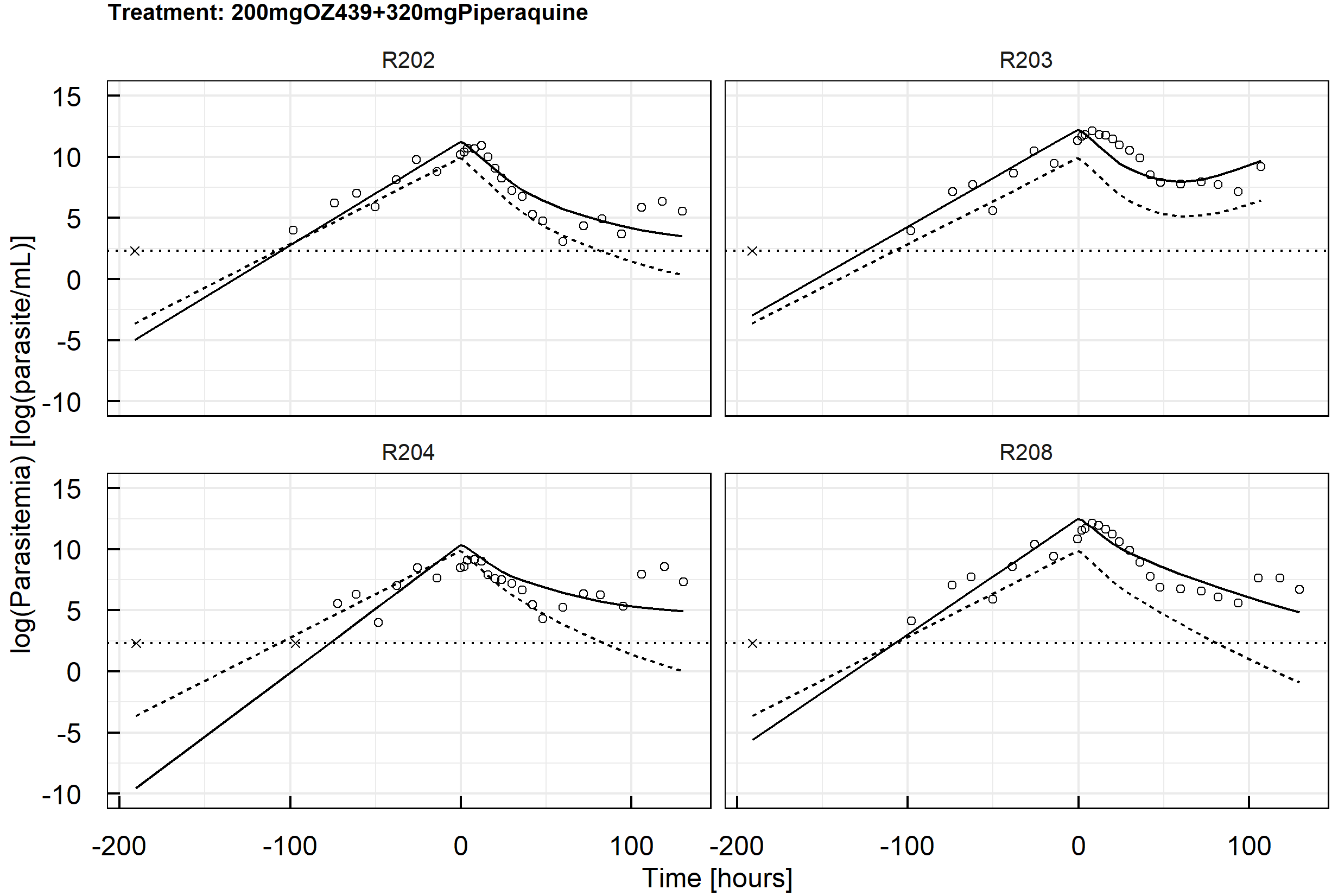


log = natural logarithm.

**B.**


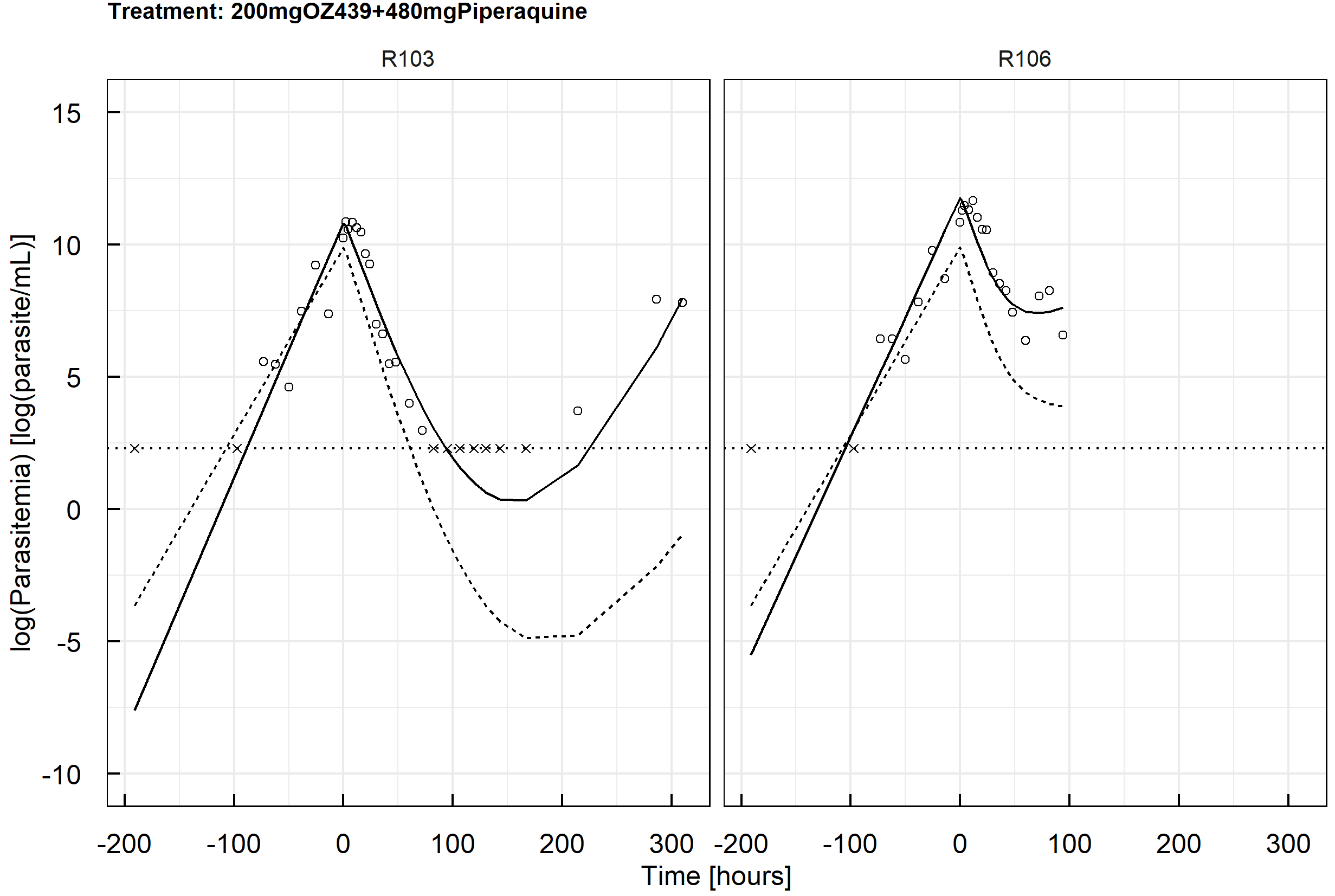


log = natural logarithm.

**C.**


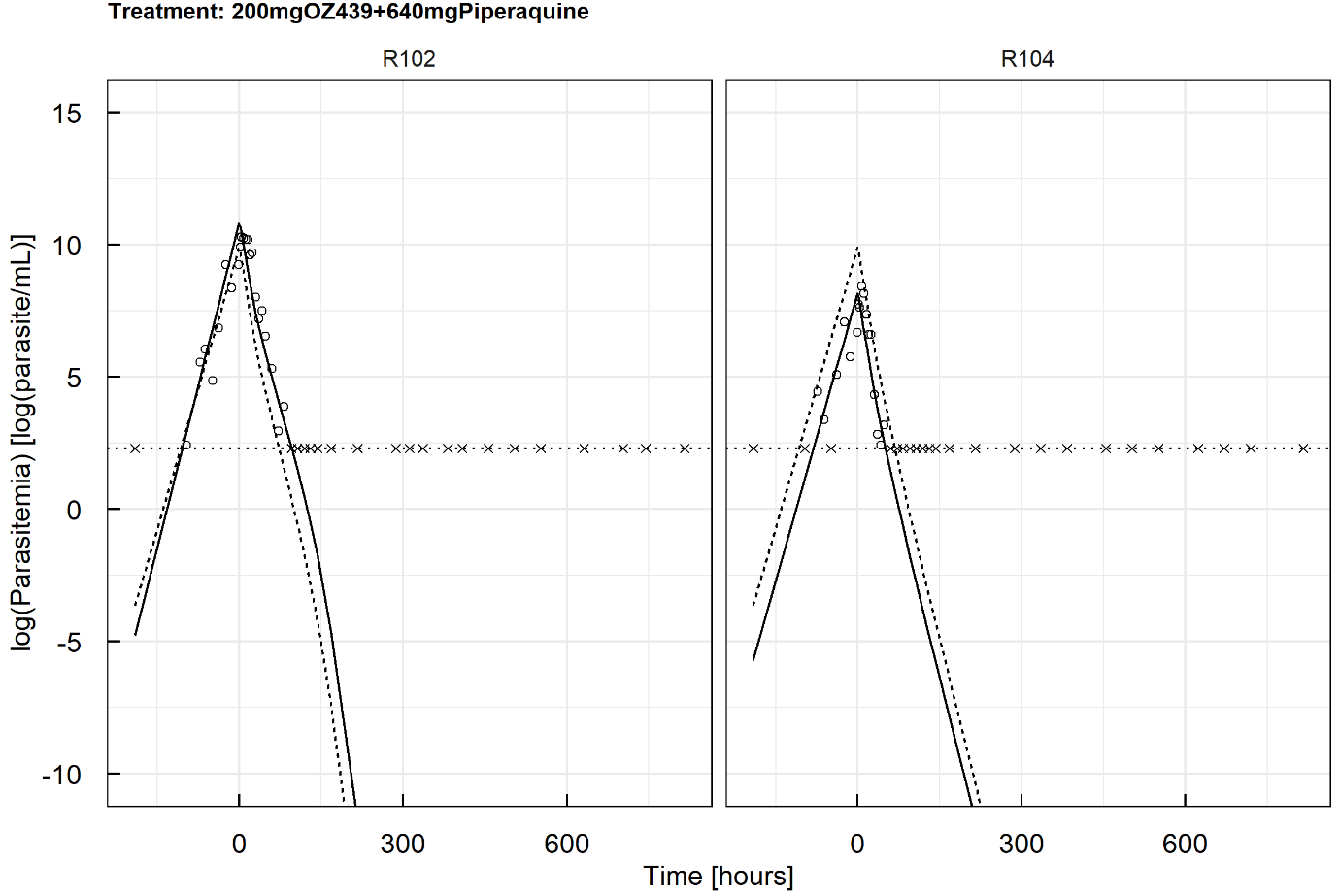


log = natural logarithm.

**D.**


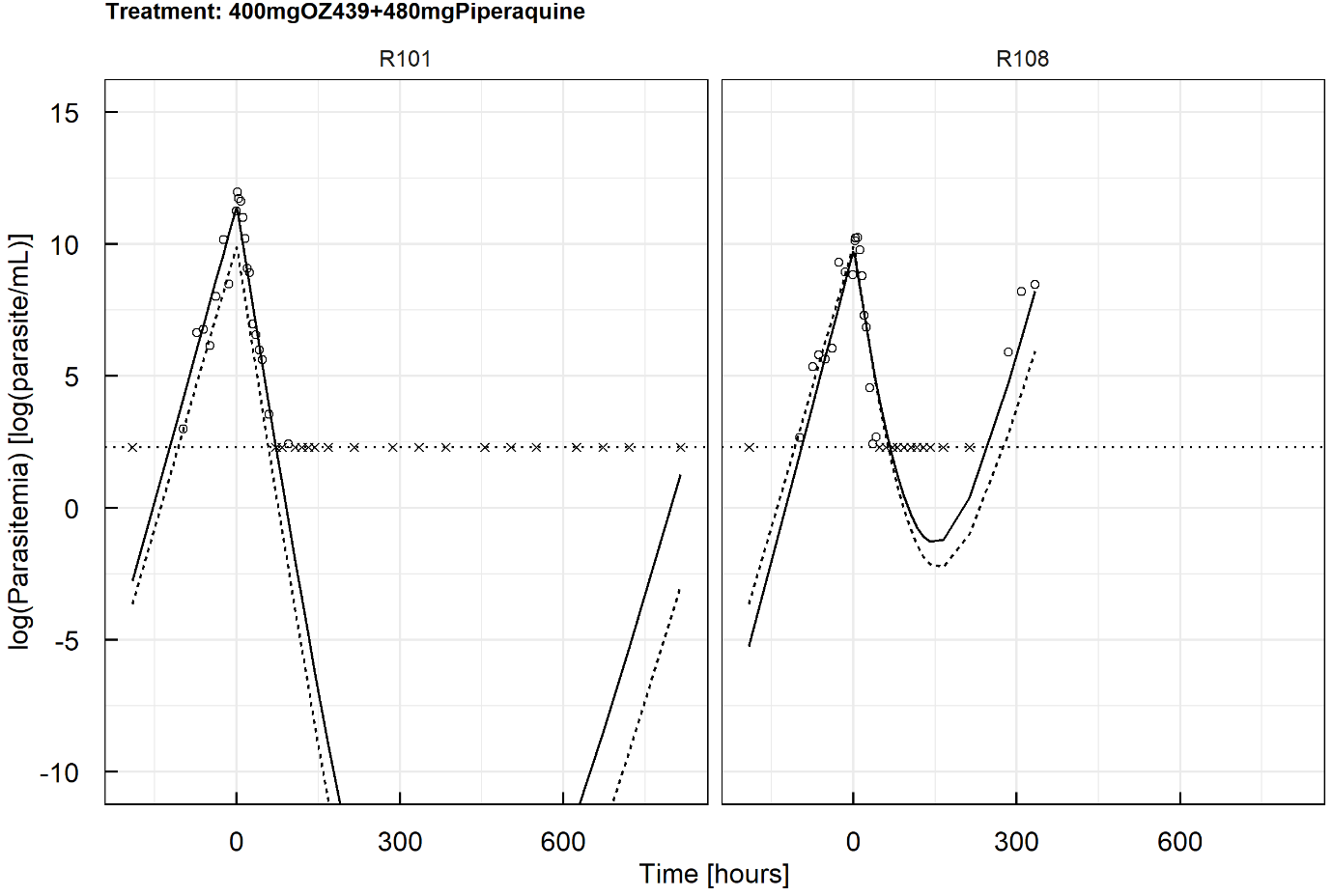


log = natural logarithm.

**E.**


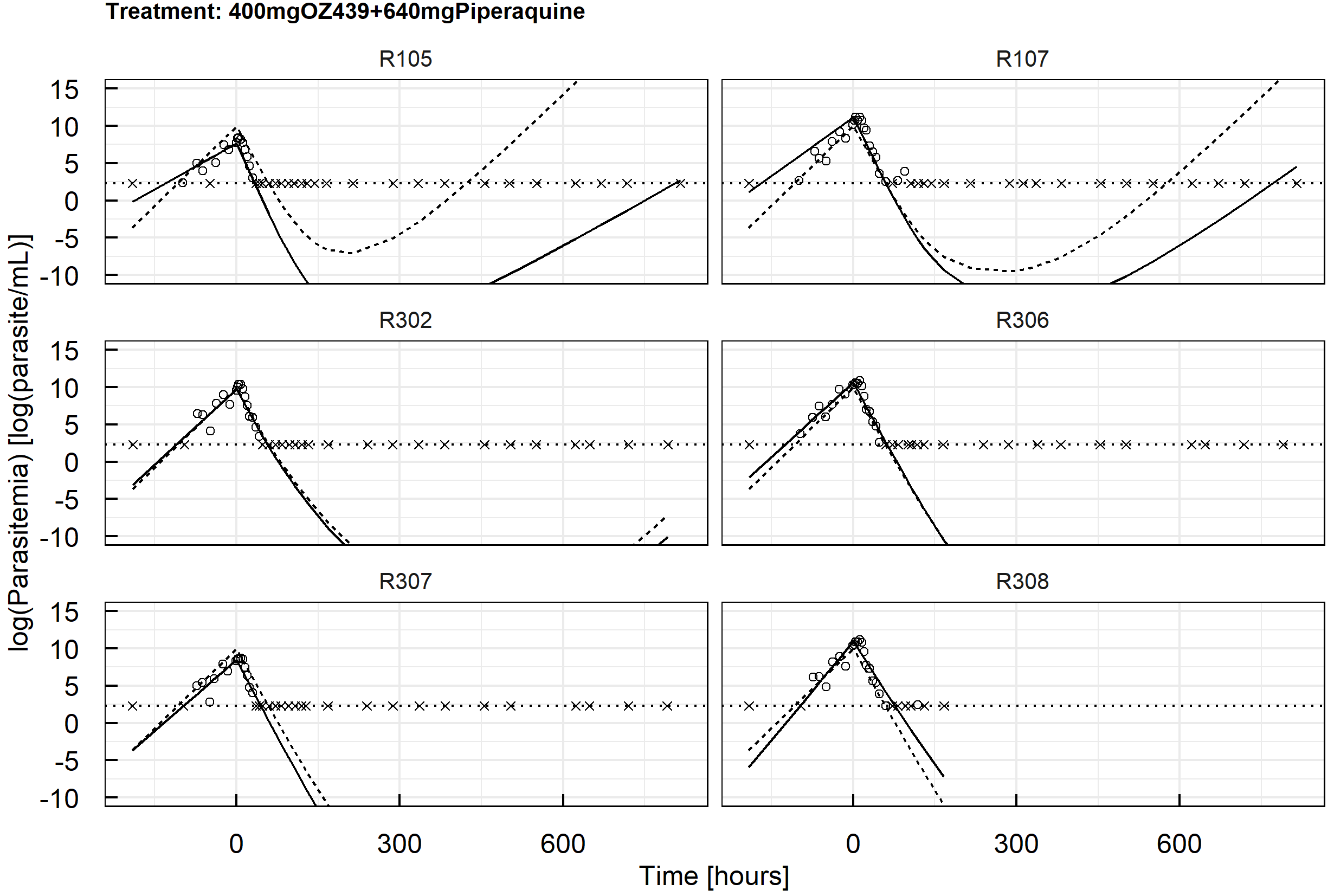


log = natural logarithm.

**F.**


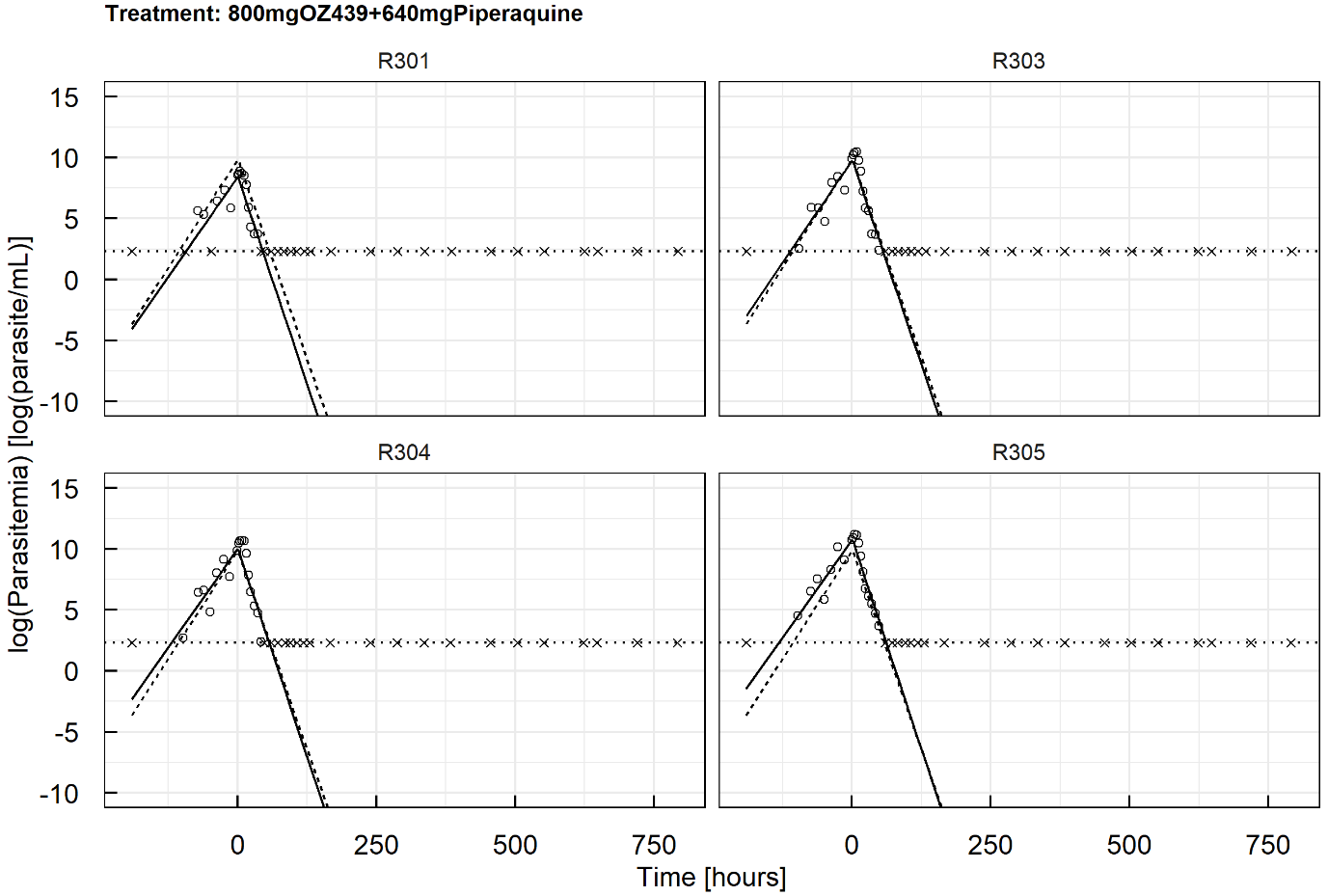


log = natural logarithm.

**G.**


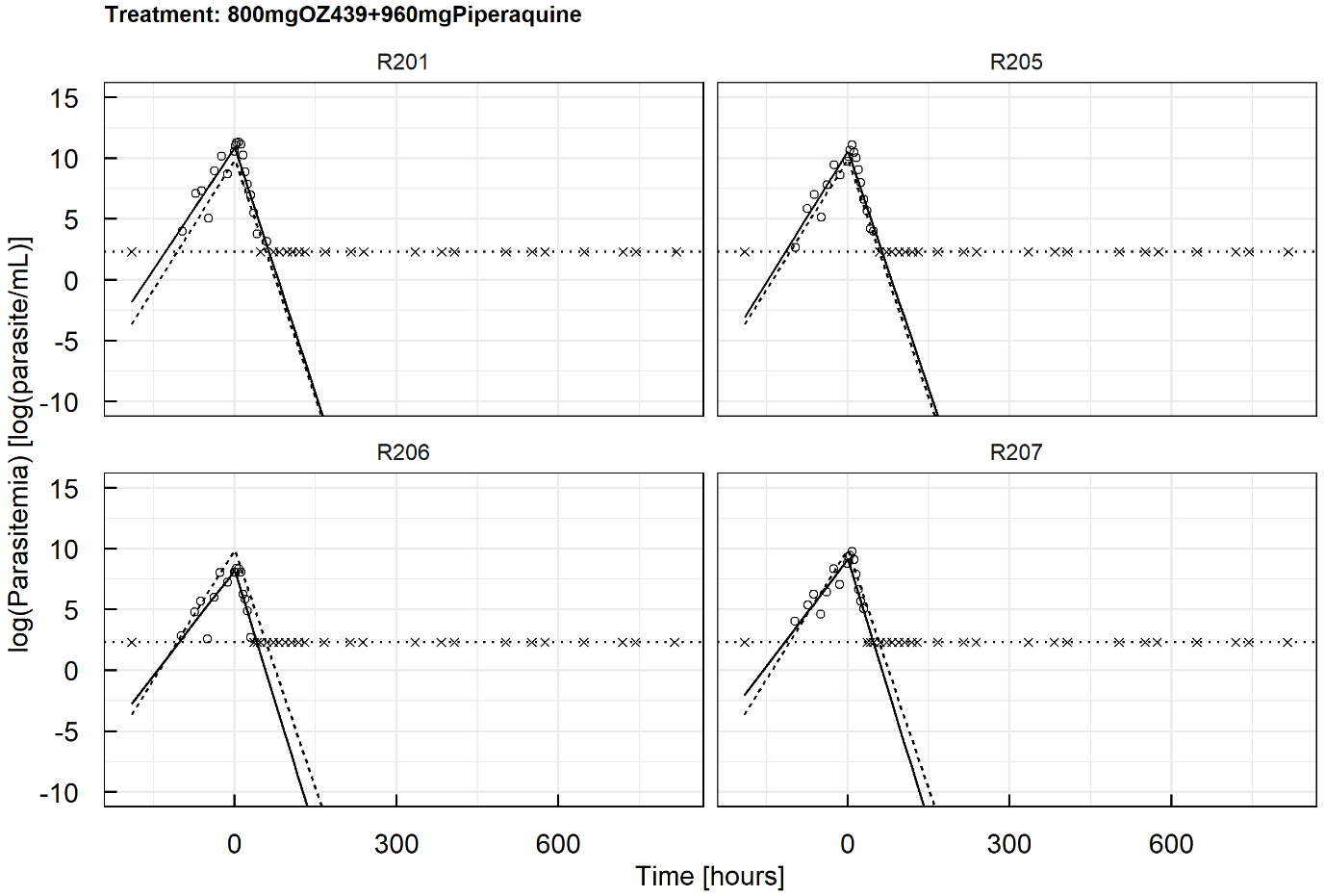


log = natural logarithm.

**Figure S7. Artefenomel (OZ439) plasma concentration-time profiles by body weight of patients in the phase 2b trial compared to the PK model built from VIS data**


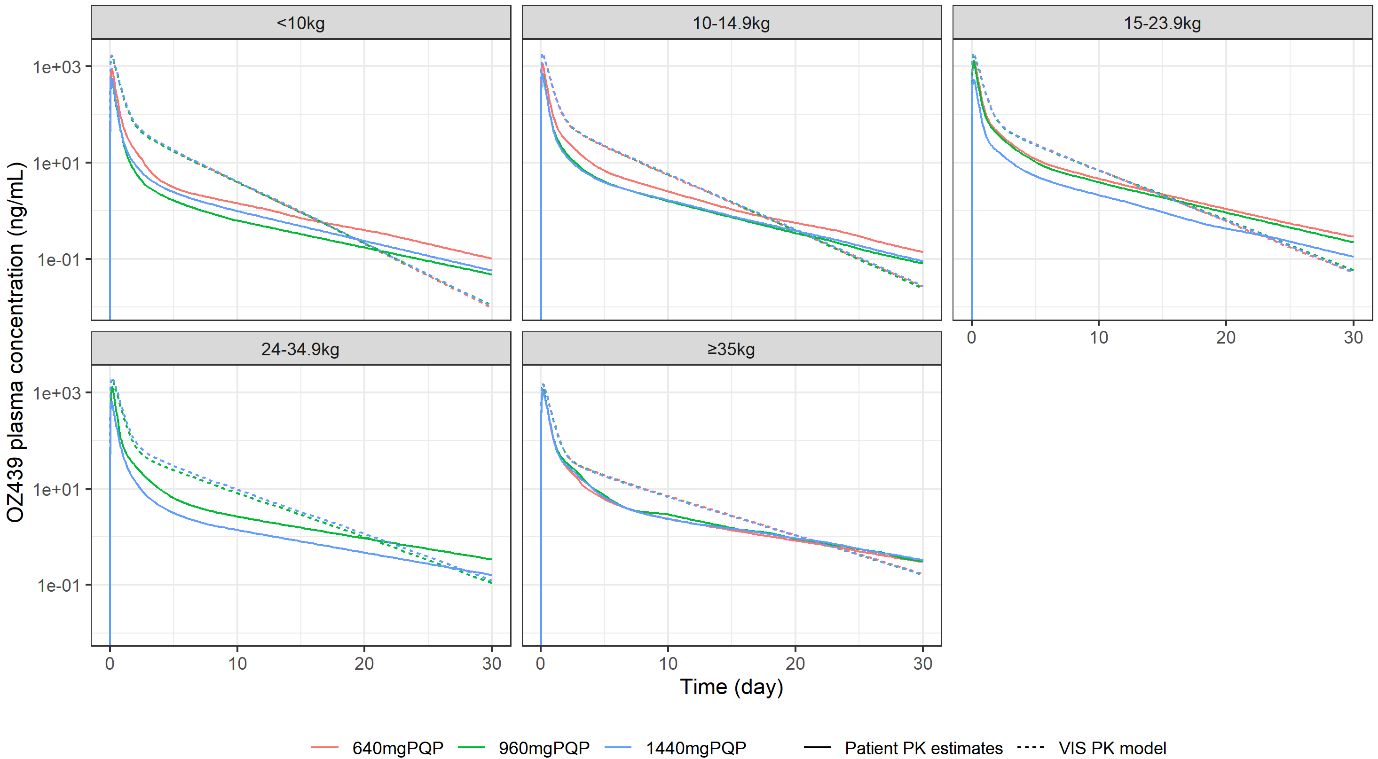


**Figure S8. Piperaquine plasma concentration-time profiles by body weight of patients in the phase 2b trial compared with the PK model built from VIS data**


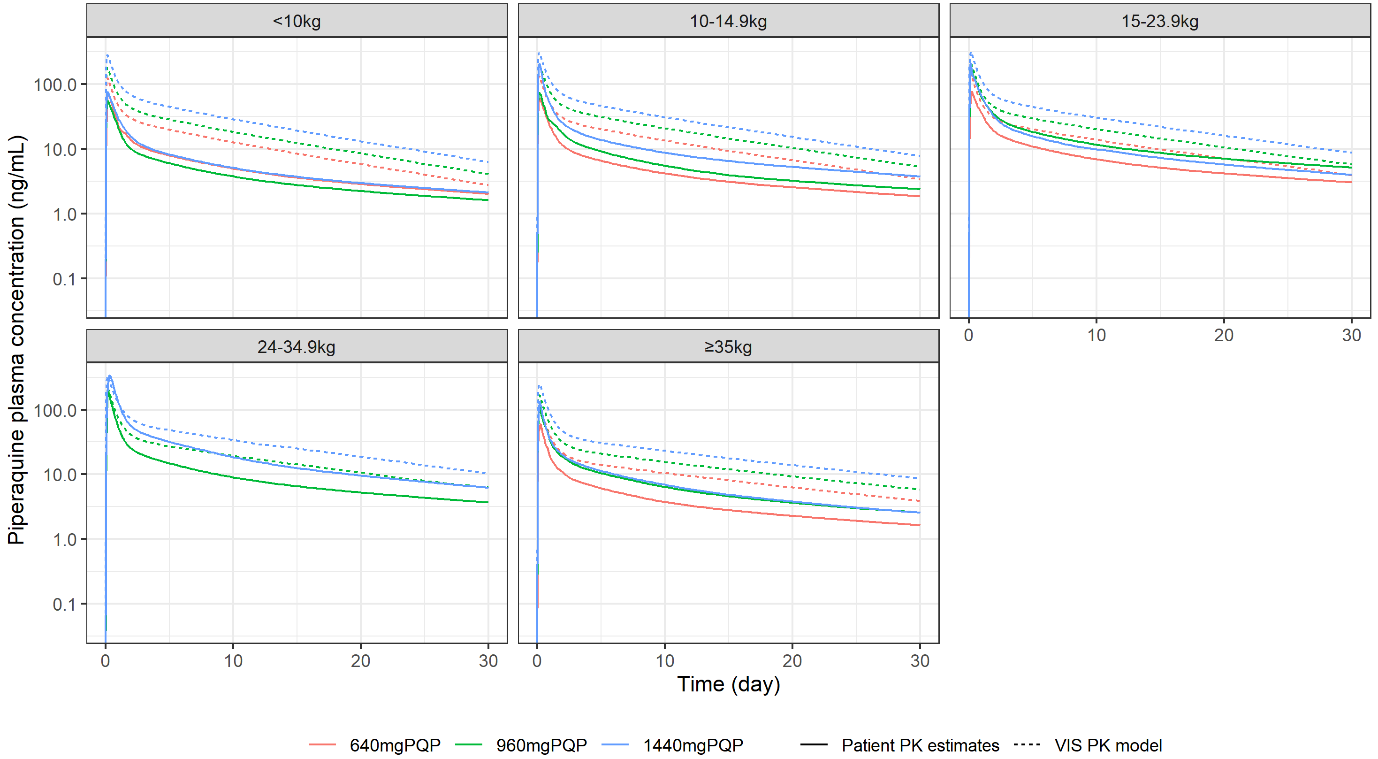


**Figure S9. Parasite killing rate as a function of artefenomel (OZ439) and piperaquine (PQP) concentration**

**
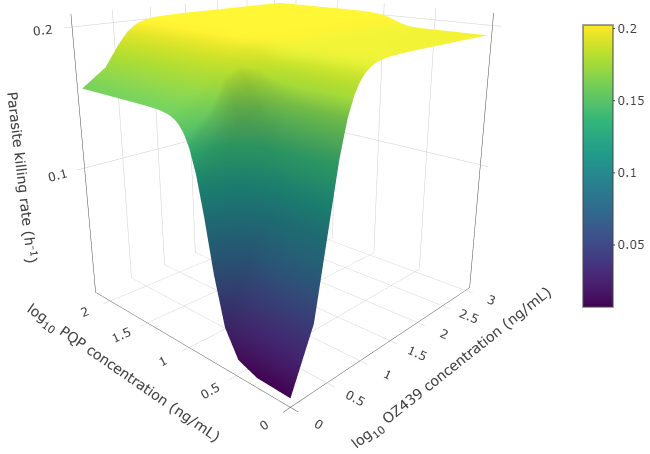
**

Combined effects (i.e. parasite killing rates) of the simulated median concentrations of artefenomel and piperaquine (based on the individual patients’ PK Bayes estimates from phase 2b study) and PD and interaction parameters estimated from the VIS data.
